# Posttraumatic bronchobiliary fistulae due to foreign body remnants after  a road traffic injury: a case report

**DOI:** 10.1186/s13256-021-02859-6

**Published:** 2021-05-22

**Authors:** Seyed-ahmad Seyed-alagheband, Mohammad-kazem Shahmoradi, Ramin Shekouhi

**Affiliations:** 1 Department of Surgery, Lorestan University of Medical Sciences, Lorestan, Iran; 2 Colorectal Research Center, Department of Surgery, Shiraz University of Medical Sciences, Zand Avenue, P.O. Box 71345-1744, Shiraz, Iran

**Keywords:** Posttraumatic, Bronchobiliary fistulae, Hepatobiliary fistulae, Case report

## Abstract

**Background:**

Bronchobiliary fistula is an extremely rare disease that involves abnormal communication between a hepatic segment and bronchial tree. It is mostly caused by untreated hydatid cyst, liver abscess, iatrogenic stenosis, and, rarely, trauma.

**Case presentation:**

We experienced an extremely rare case of bronchobiliary fistula after motor vehicle accident. A 15-year-old Persian boy visited our clinic with chief complaints of persistent pleuritic chest pain, productive cough, weight loss, and fever for 2 months. Coronavirus disease 2019 reverse transcription polymerase chain reaction test was negative. Chest X-ray revealed hazy opacification of right lower lobe. Bronchoalveolar lavage for acid-fast bacillus came back negative. Thoracoabdominal computed tomography scan revealed a collection in segment VIII of the liver communicating with another 13 × 5 cm multiloculated collection in the lower lobe of the right lung, with air foci within the collection. Right posterolateral thoracotomy was performed with the impression of bronchobiliary fistula. Drainage of hepatic collection with debridement, diaphragmatic repair, and open decortication of lung followed by resection of the involved segment of the right lung was performed. Histopathologic evaluations revealed abscess formation in pulmonary tissue, and many multinucleated giant cells were seen that appear to be due to foreign body remnants after previous laparotomy surgery. The foreign body seemed to be the remnants of Surgicel absorbable hemostat.

**Conclusions:**

Herein, we report an extremely rare case of a posttraumatic bronchobiliary fistula caused by remnants of Surgicel hemostatic agent. Bronchobiliary fistula is mainly caused by untreated hydatid cyst, liver abscess, iatrogenic stenosis, and, rarely, trauma. Migration and erosion of oxidized regenerated cellulose through the diaphragm seems to be the causative factor of bronchobiliary fistula in this patient.

## Introduction

Bronchobiliary fistula (BBF) is an extremely rare disease first introduced by Peacock in 1850 [1]. BBF is an abnormal communication between a hepatic segment and bronchial tree. It is mostly caused by untreated hydatid cyst, liver abscess, iatrogenic stenosis, and, rarely, trauma [2]. Herein, we describe a rare case of BBF after motor vehicle accident (MVA) and blunt abdominal trauma. Our patient differs in that the BBF seems to be caused by remnants of nonabsorbed Surgicel (an absorbable hemostatic agent) particles in the abdominal cavity after laparotomy surgery.

## Case presentation

A 15-year-old Persian boy was involved in a car accident 3 months prior. He underwent chest tube insertion owing to massive right-sided pneumothorax. The patient was taken to operation room (OR) for exploratory laparotomy for blunt trauma of abdomen. Surgery revealed a grade 3 liver laceration in segments VI–VII with massive hemoperitoneum. Pringle maneuver was performed to reduce bleeding from the liver. Furthermore, the laceration in segment VI was repaired with deep mattress sutures. However, the surgical team used an absorbable hemostatic agent (Surgicel) in the liver VII segment owing to severe liver contusion caused by blunt trauma to the abdomen. The Surgicel was placed in the anterior part of the liver VII segment, definitive hepatic repair was performed, and he was discharged 10 days later. However, the patient was referred to the clinic with complaints of persistent pleuritic chest pain, productive cough, weight loss, and fever for the last 2 months.

**Fig. 1:**
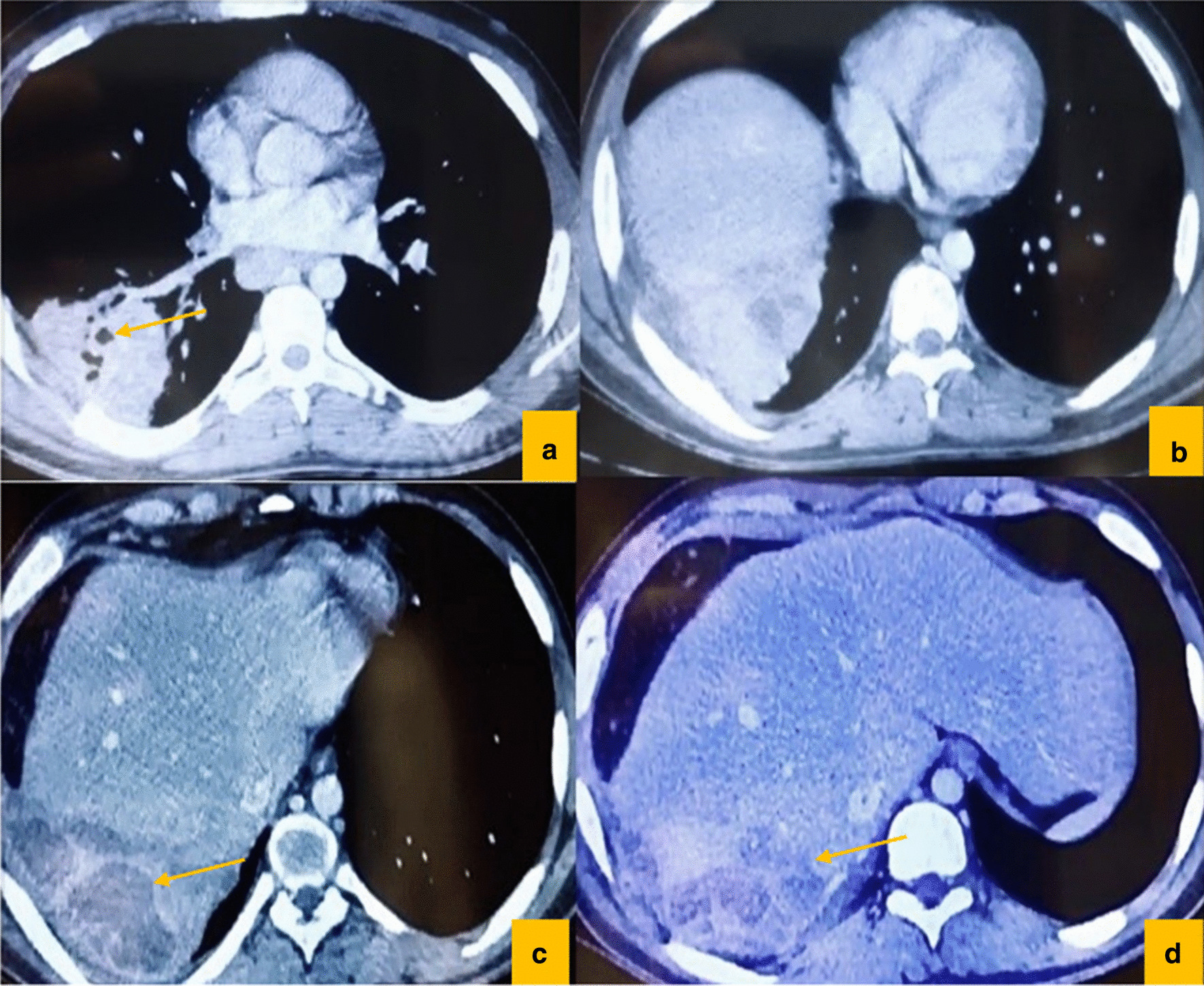
CT scan of thoracoabdominal region; arrow showing the right lung collection with air foci within the collection (**a**); arrow indicating the collection in segment VIII of liver (**c**) and (**d**)

Upon arrival, the patient was hemodynamically stable without signs of respiratory distress. Past medical history was unremarkable except for the recent surgery due to MVA. Family history was unremarkable. Physical examination revealed mild right upper quadrant (RUQ) tenderness at site of previous surgery. Lung auscultation showed decreased breath sounds at the base of right lung. Laboratory data showed leukocytosis (WBC count 14,000) with marked elevation in the neutrophil count, and high C- reactive protein (CRP) levels. Coronavirus disease 2019 (COVID-19) reverse transcription polymerase chain reaction (RT-PCR) test was negative. Chest X-ray revealed hazy opacification of the right lower lobe. Bronchoalveolar lavage (BAL) for acid-fast bacillus (AFB) came back negative. Thoracoabdominal computed tomography (CT) scan revealed a collection in segment VIII of the liver communicating with another 13 × 5 cm multiloculated collection in the lower lobe of the right lung, with air foci within the collection (Fig. 1). After thoracoabdominal CT scan, sputum analysis was performed with suspicion of BBF. However, there was no sign of bile in the sputum.

### Treatment

The patient was scheduled for thoracotomy, and preoperative management was considered.

**Fig. 2:**
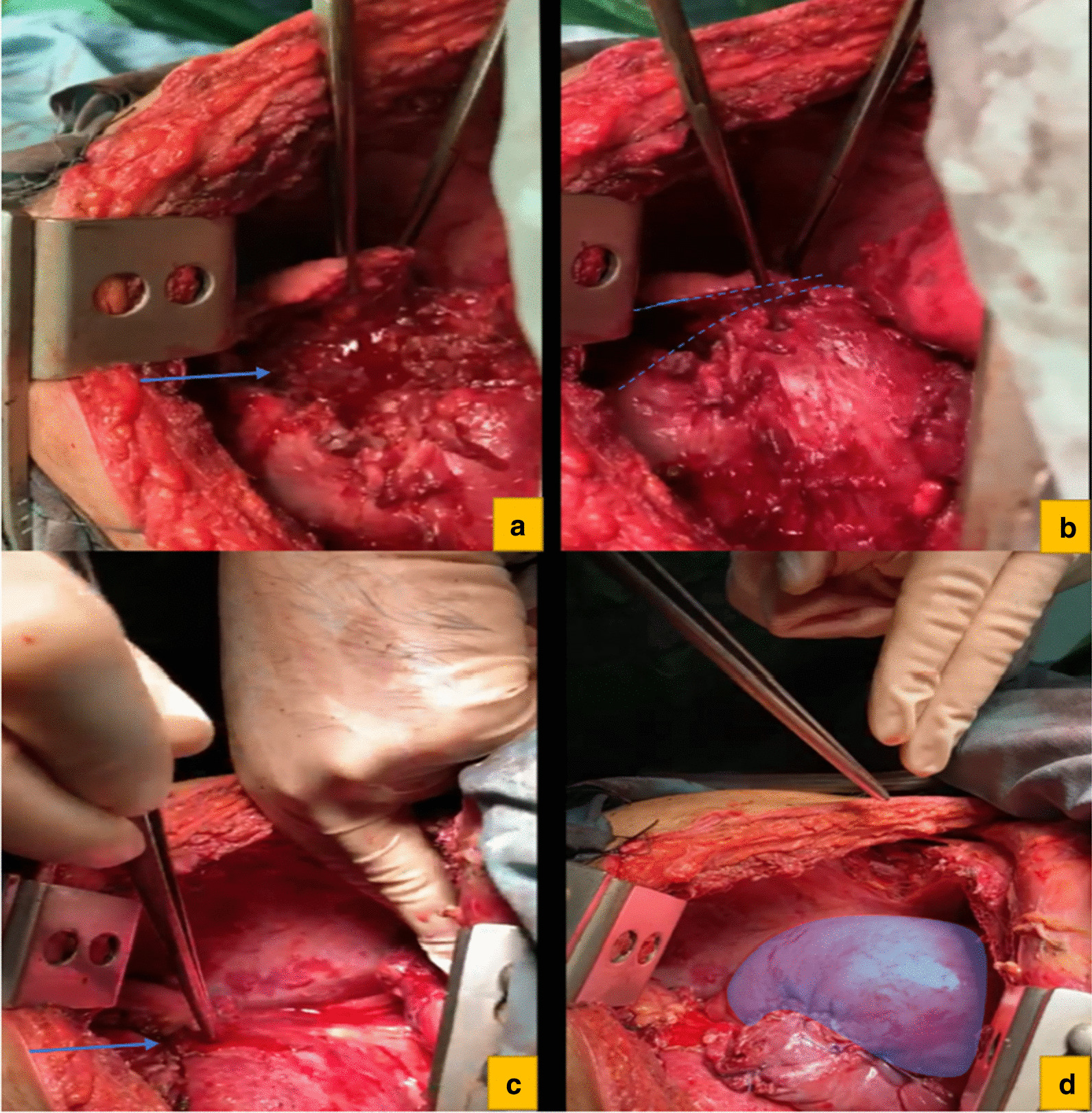
Right posterolateral thoracotomy, and transdiaphragmatic approach for drainage of liver collection. Ruptured diaphragm due to BBF shown by blue arrow (**a**); repaired diaphragm after the operation and drainage of liver collection indicated by arrow (**c**); and lung’s right middle lobe after fistulectomy and segmentectomy of involved right lower lobe (**d**)

**Fig. 3:**
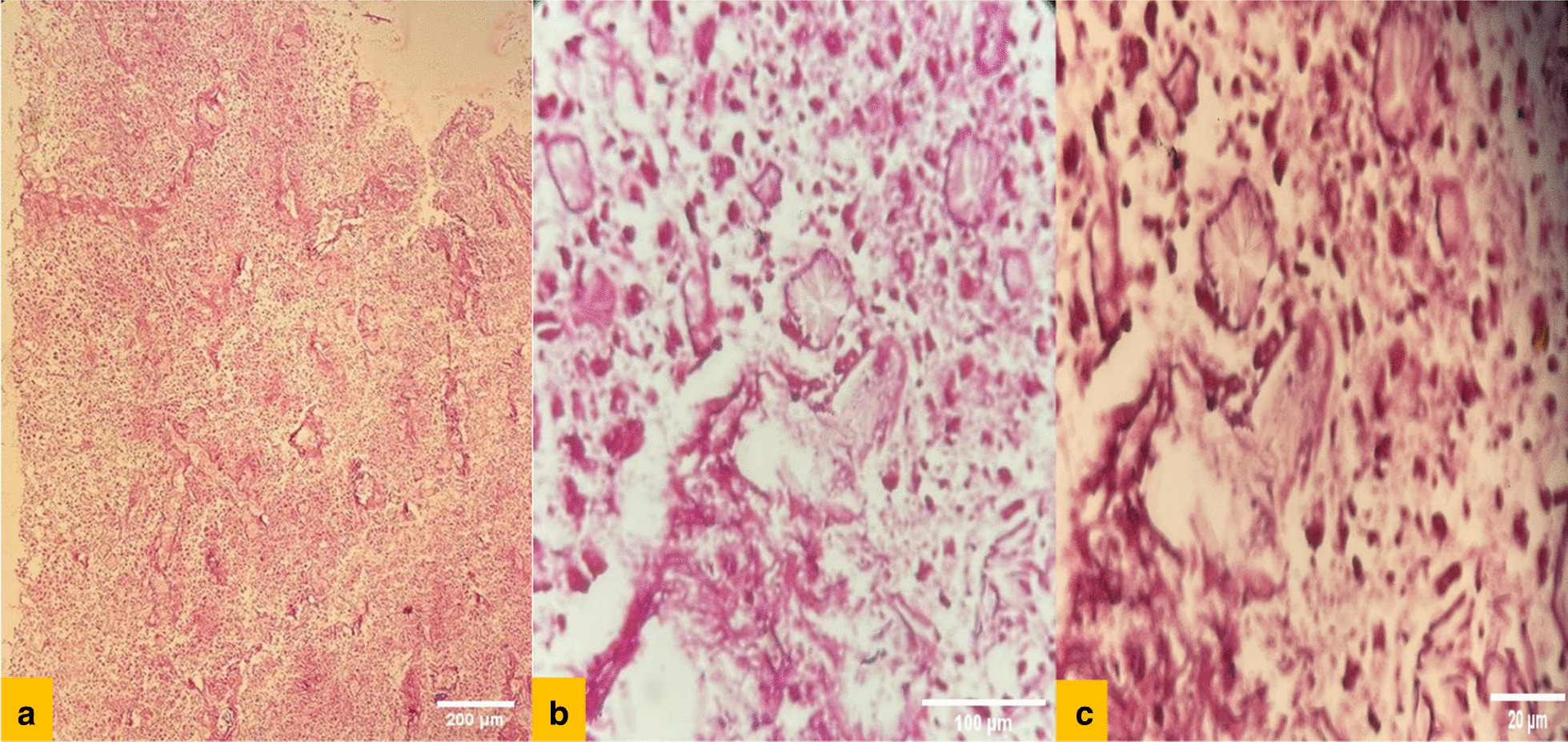
Histology of nonabsorbed Surgicel particles found in excised specimen of BBF

**Fig. 4.**
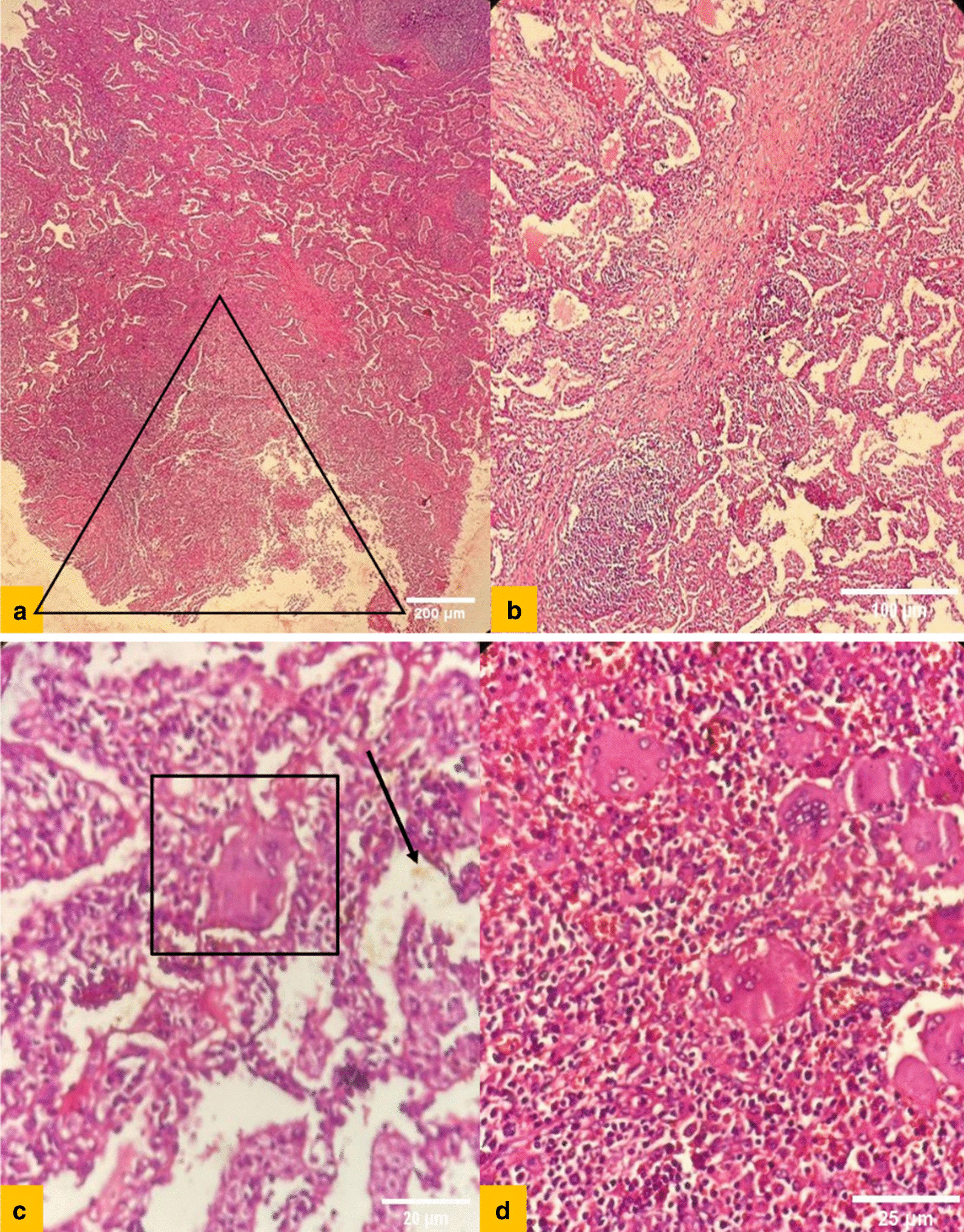
Histologic evaluation of the specimen; fistulation of BBF into the lung parenchyma (×4) (**a**); diffuse fibrosis in pulmonary tissue (×10) (**b**); giant cell within the involved lung segment with, interestingly, bile staining in the alveoli (black arrow) (×40) (**c**); many multinucleated giant cells in most parts of involved liver tissue that appear to be due to foreign body remnants (Surgicel) after previous surgery (×40) (**d**)

The surgery was conducted under general anesthesia. The patient was scheduled for thoracotomy with the purpose of complete drainage of lung abscess and resection of involved segments. In addition, a transdiaphragmatic approach was carried out for drainage of liver collection (Fig. 2). Diffuse pleural thickening and parenchymal adhesion bands with collection formation were revealed during the operation. Drainage of hepatic collection with debridement and diaphragmatic repair were done. Furthermore, open decortication of lung followed by resection of involved segment of right lung was performed. Intercostal chest drains were placed. Histopathologic evaluation revealed abscess formation in pulmonary tissue with diffuse fibrosis. Many multinucleated giant cells were seen in most parts of inflamed tissues, which appeared to be due to remnants of nonabsorbed Surgicel (an absorbable hemostatic agent) particles used in previous laparotomy surgery (Figs. 3 and 4).

### Outcome and follow-up

The postoperative course was uneventful without any signs of short-term complications. He had no episodes of cough, fever, and bilioptysis after the surgery. The patient was discharged 4 days after surgery with the abdominal drain. The abdominal drain had 10 mL output after 1 week. On the second follow-up visit 14 days postoperation, abdominal ultrasound was unremarkable without any collection, and the drain was removed.

## Discussion

Classically, BBF can be divided into congenital and acquired. Congenital BBF (CBBF) mostly presents early in life with poor feeding, bilioptysis, and respiratory distress. It mainly coexists with other biliary tract anomalies [3]. Acquired BBF can be a result of damage to the bronchioles, diaphragm, or biliary tracts [4]. Hydatid cysts have been known to be the main cause of BBF. However, thanks to use of broad-spectrum antibiotics and early detection, incidence has decreased tremendously. Other causes of BBF include subphrenic abscess formation, malignancy, iatrogenic factors, and trauma. Posttraumatic BBF is extremely rare, with few cases reported worldwide [5]. To the best of our knowledge, there are only 20 cases (including this patient) of published posttraumatic BBF in English literature (Table 1). Among a total of 20 patients, 17 (85%) patients were males and the rest were females, with a mean age of 25.0 years. Penetrating injures, mostly by gunshot, were the most common cause of posttraumatic BBF (60%). Postural intractable cough, bilioptysis (bile in the sputum), fever, and pleuritic chest pain are the usual presentation of BBF [6]. Bilioptysis ranges from mild bile-streak sputum to expectoration of extensive amounts of bile [7].

**Table 1:** Published cases of BBF

Year	Age	Sex	Bilioptysis	Lobe involvement	Mechanism of trauma	Surgery	Survival
1983 [22]	29	M	Yes	Right	Penetrating trauma (gunshot)	Thoracotomy, lobe resection	A
1984 [23]	38	M	Yes	Right	Penetrating trauma (gunshot)	Thoracotomy, lobe resection	A
1994 [24]	15	M	Yes	Right	Penetrating trauma (gunshot)	Thoracotomy	A
2002 [25]	26	M	Yes	NM	NM	Thoracotomy, ERCP	A
2002 [25]	42	M	No	NM	NM	ERCP	A
2002 [25]	33	M	No	NM	NM	ERCP	A
2007 [26]	20	M	Yes	Right	Penetrating trauma (gunshot)	Thoracotomy, lobe resection	A
2009 [9]	18	M	Yes	Right	Blunt trauma (MVA)	Thoracotomy	A
2009 [27]	18	F	Yes	Right	Penetrating trauma (gunshot)	ERCP	A
2009 [28]	27	M	Yes	Right	Penetrating trauma (gunshot)	Thoracotomy	A
2012 [8]	NM	M	Yes	NM	Penetrating trauma (gunshot)	Thoracotomy, ERCP	A
2012 [8]	NM	M	NM	NM	Penetrating trauma (stab wound)	Thoracotomy, ERCP	A
2012 [8]	NM	M	NM	NM	Penetrating trauma (gunshot)	Thoracotomy	A
2012 [8]	NM	M	NM	NM	Penetrating trauma (gunshot)	Thoracotomy, ERCP	A
2012 [8]	NM	F	NM	NM	Penetrating trauma (gunshot)	Thoracotomy, ERCP	A
2012 [29]	NM	F	Yes	Right	Blunt trauma (MVA)	Laparotomy, ERCP, PTC, embolization of fistula tract, Thoracotomy 1 year later	A
2014 [30]	29	M	Yes	Right	Blunt trauma	ERCP, stent insertion, fistula blockage with cyanoacrylate glue and endocoils	A
2018 [31]	18	M	Yes	Right	Blunt trauma (falling down)	ERCP, stent insertion	A
2020 [32]	22	M	Yes	Right	Penetrating trauma (gunshot)	Laparotomy, ERCP, PTC, thoracotomy, fistulectomy, lobe resection	A
2021 (our case)	15	M	No	Right	Blunt trauma (MVA)	Thoracotomy, fistulectomy, lobe resection	A

*ERCP*, endoscopic retrograde cholangiopancreatogram; *PTC*, percutaneous transhepatic cholangiography; *MVA*, motor vehicle accident; *A*, alive

BBF can be initially misdiagnosed as acquired pneumonia, making the diagnosis of BBF difficult. In terms of diagnosis, chest X-ray may reveal pleural effusion and lung collapse. CT scan shows any fluid collection, including subphrenic abscess, damage to inferior lobes, and obstructed biliary tracts. Pleural fluid and sputum analysis should be assessed for elevated bilirubin levels; however, it requires a high index of suspicion [8]. Santra *et al.* [9] recommended hepatobiliary iminodiacetic acid (HIDA) scan as a precise noninvasive diagnostic tool for BBF. However, Nigro *et al.* [10] stated that, owing to reversal of transdiaphragmatic pressure gradient in patients on mechanical ventilation, HIDA scan falls short of confirming the diagnosis.

Magnetic resonance cholangiography (MRCP), ERCP, or percutaneous transhepatic cholangiography (PTCD) are the safest, most precise nonsurgical interventions that can be successfully operated [8]. ERCP is more practical than MRCP because of its capacity to identify the BBF precisely, and it also has potential for therapeutic interventions [11]. ERCP is considered the first-line treatment in BBF. Traditionally, surgery was the keystone of treatment. However, recently, more conservative, noninvasive treatment methods are preferred initially. Accordingly, among the reports of 20 patients with BBF published in English literature, ERCP was performed in 12 (60%) patients with the goal of complete drainage of hepatic collection. ERCP-guided biliary decompression and stent insertion facilitate bilious drainage into the gastrointestinal tract and successful resolution of BBF. Despite its noninvasive nature, complications including bleeding, cholangitis, pancreatitis, duodenal perforation, and stent occlusion occur. Although ERCP showed success in treatment of BBF, the failure rate of conservative methods alone without surgical interventions was estimated at nearly 38% [12]. According to previous case reports, death associated with posttraumatic BBF has not been reported.

Open surgery is indicated if conservative techniques fail. Multiple surgical approaches have been recommended for treatment of BBF, including thoracotomy, thoracoabdominal approach, and laparotomy. Most studies preferred the thoracoabdominal approach as the definite surgical method. According to our results, 15 (75%) patients, underwent thoracotomy for resection of involved lung segment, and fistulectomy (Table 1). It provides accessibility to perform decortication, complete excision of fistulae tract, wedge resection of lung parenchyma, lobectomy, and diaphragmatic closure [13, 14]. We considered surgical intervention as the first-line therapy because of the subphrenic abscess and better management of biliary leakage to the abdominal cavity. Our case differs in that the BBF seems to be caused by remnants of nonabsorbed Surgicel particles in the abdominal cavity after laparotomy surgery.

Surgicel, which is a bioabsorbable hemostatic agent, consists of oxidized regenerated cellulose. It is a thrombogenic material mainly used to control bleeding. Once the Surgicel is placed in the surgical bed, it forms a gelatinous mass that helps control the bleeding and clot formation [15]. Macrophage processing seems to play a role in the absorption mechanism of Surgicel in the body; however, the exact mechanism is not well understood. The absorption process of Surgicel by the body begins after 18 hours, and will usually be absorbed within 6 weeks [16]. However, in our case, Surgicel was not completely absorbed after 3 months of insertion, as we noticed remnants of Surgicel on histological evaluation.

Although oxidized cellulose is a safe hemostat, complications including delayed absorption, granuloma formation, and compression effect to surrounding organs still occur. There have been multiple reports of paraplegia following thoracotomy due to compressive effects of oxidized cellulose. Swelling and migration of oxidized cellulose through intervertebral foramen into the spinal canal were the main cause of paraplegia following Surgicel insertion after thoracotomy. The pressure gradient between the spinal canal and pleural space seems to be the causative factor for postthoracotomy Surgicel migration [17–20].

In addition, Dokumcu *et al.* [21] reported a patient of esophageal atresia (EA) with distal tracheoesophageal fistula (TEF) who underwent thoracotomy for surgical management of esophagus and tracheal defects. However, 1 month after thoracotomy, the patient developed symptoms indicative of TEF. Esophagoscopy revealed patent TEF with the nonabsorbed Surgicel in the TEF lumen, which was not removed to prevent fistulae enlargement. Three weeks later, the Surgicel hemostat disappeared and the patient underwent another thoracotomy for closure of TEF. Dokumcu *et al.* concluded that postoperative Surgicel migration and expansion were the cause of TEF recurrence.

In our case, the major cause of Surgicel migration, and erosion through the diaphragm, seems to be the increased thoracoabdominal pressure gradient (TAPG), which caused the migration of Surgicel to reach the diaphragm, especially in the setting of increased intraabdominal pressure following blunt trauma and laparotomy surgery. That said, our second hypothesis is that the delayed absorption of Surgicel material could have caused an abscess within the involved liver segment, which later on the abscess fistulized to the adjustment diaphragm and lung tissue, causing BBF. Regardless, delayed absorption of Surgicel hemostatic agent seems to be the main cause in the formation of BBF. However, due to rarity of the disease, there is not enough evidence to support any of these hypotheses. To the best of our knowledge, this is the first case of posttraumatic BBF due to remnants of nonabsorbed Surgicel hemostatic agent.

## Conclusion

Herein, we reported an extremely rare case of posttraumatic bronchobiliary fistula (BBF) caused by remnants of Surgicel hemostatic agent. BBF is mainly caused by untreated hydatid cyst, liver abscess, iatrogenic stenosis, and, rarely, trauma. The patient was a 15-year-old boy who developed BBF following laparotomy surgery, in which Surgicel hemostatic agent was inserted to control bleeding due to liver laceration. Migration and erosion of oxidized regenerated cellulose through the diaphragm seems to be the causative factor of BBF in this patient. We reviewed the early signs and symptoms of BBF, the gold standard diagnostic method, and emphasized the importance of surgical management in the treatment of BBF.

### Patient perspective

Five months ago, I had an accident and I was rushed to a nearby hospital. I had an operation and was admitted for two weeks. 2 days after I discharged from the hospital, I developed with persistent cough, fever and malaise. First, I thought I was infected with covid-19 due to my recent hospital admission. However, my COVID-19 test came back negative. After a lot of investigations, I was diagnosed with BBF. I underwent a second surgery and I was doing well. All of my symptoms resolved and I am grateful that I can go back to my normal life.

## Data Availability

All data generated or analyzed during this study are included in this published article.
